# *ZmSTK1* and *ZmSTK2*, encoding receptor‐like cytoplasmic kinase, are involved in maize pollen development with additive effect

**DOI:** 10.1111/pbi.12880

**Published:** 2018-02-13

**Authors:** Mingxia Fan, Chunyu Zhang, Lei Shi, Chen Liu, Wenjuan Ma, Meiming Chen, Kuichen Liu, Fengchun Cai, Guohong Wang, Zhengyi Wei, Min Jiang, Zaochang Liu, Ansar Javeed, Feng Lin

**Affiliations:** ^1^ College of Bioscience and Biotechnology Shenyang Agricultural University Shenyang Liaoning China; ^2^ Corn Research Institute Liaoning Academy of Agricultural Sciences Shenyang Liaoning China; ^3^ Laboratory of Plant Bioreactor and Genetics Engineering Jilin Provincial Key Laboratory of Agricultural Biotechnology Agro‐Biotechnology Research Institute Jilin Academy of Agricultural Sciences Jilin Changchun China; ^4^ Shanghai Agrobiological Gene Center Shanghai Academy of Agricultural Sciences Shanghai China

**Keywords:** *Zea mays*, receptor‐like cytoplasmic kinase, pollen development, additive effect, male gametophyte transmission, protein interaction

## Abstract

Pollen germination and pollen tube growth are important physiological processes of sexual reproduction of plants and also are involved in signal transduction. Our previous study reveals that ZmSTK1 and ZmSTK2 are two receptor‐like cytoplasmic kinases (RLCK) homologs in *Zea mays* as members of receptor‐like protein kinase (RLK) subfamily, sharing 86% identity at the amino acid level. Here, we report that *ZmSTK1* and *ZmSTK2*, expressed at late stages of pollen development, regulate maize pollen development with additive effect. *ZmSTK1* or *ZmSTK2* mutation exhibited severe pollen transmission deficiency, which thus influenced pollen fertility. Moreover, the kinase domains of ZmSTKs were cross‐interacted with C‐terminus of enolases detected by co‐immunoprecipitation (Co‐IP) and yeast two‐hybrid system (Y2H), respectively. Further, the detective *ZmSTK1* or *ZmSTK2* was associated with decreased activity of enolases and also reduced downstream metabolite contents, which enolases are involved in glycolytic pathway, such as phosphoenolpyruvate (PEP), pyruvate, ADP/ATP, starch, glucose, sucrose and fructose. This study reveals that *ZmSTK1* and *ZmSTK2* regulate maize pollen development and indirectly participate in glycolytic pathway.

## Introduction

Pollen development is a complex and highly coordinated biological process. Two distinct and successive developmental phases, microsporogenesis and microgametogenesis, lead to the production of mature pollen grains. In *Zea mays*, mature microspores/pollens emerge after meiosis followed by two cycles of mitosis and consist of two sperm cells and one vegetative cell. While the two smaller sperm cells participate in the double fertilization and finally produce zygote, endosperm and seed tissues, the vegetative cell provides nutrition for pollen germination and pollen tube elongation.

Pollen development involves regulation of numbers of proteins and signalling molecules (Beale and Johnson, 2013; Chen *et al*., 2014). Therefore, disruption of pollen‐specific gene expression due to genetic mutation or environmental effect regularly results in a failure of male fertility. In Arabidopsis, *ANX1* and *ANX2* are pollen‐expressed homologous genes of FERONIA receptor‐like protein kinase (FER‐RLK) family. Mutants *anx1* and *anx2* do not exhibit any phenotypes, and the pollen tubes of double homozygous *anx1 anx2* mutants fail to reach the locules of the ovary, suggesting that *ANX1* and *ANX2* function redundantly to control the timing of pollen tube discharge (Boisson‐Dernier *et al*., 2009). LIP1 and LIP2, belonging to the receptor‐like cytoplasmic kinase (RLCK) VII subfamily, also show functional redundancy to attraction towards the female attractant AtLURE1 in Arabidopsis (Liu *et al*., 2013). Highly homologous inositol polyphosphate kinases AtIPK2α and AtIPK2β in Arabidopsis act redundantly during pollen development, pollen tube guidance and embryogenesis (Zhan *et al*., 2015). Also in Arabidopsis, PIP5K1 and PIP5K2, belonging to the phosphatidylinositol 4‐phosphate 5‐kinase (PIP5 K) family, are functionally redundant as homozygous double mutants do not render viable pollen grains (Ugalde *et al*., 2016). In addition, the expression of AtSTP9, one of 14 highly homologous monosaccharide transporters, is prominent in germinating pollen tubes, while AtSTP2 are expressed at the beginning of callose degradation and microspore release from the tetrads (Schneidereit *et al*., 2003; Truernit *et al*., 1999). In summary, the previous studies show that pollen germination and pollen tube growth are usually regulated by members of the same gene family through gene function redundancy or functional differences, while genes with additive effect involving pollen development are almost unknown.

Receptor‐like protein kinases (RLKs) pertain to a large protein subfamily that can transmit ligand signals through autophosphorylation and initiate a signal cascade reaction (Salem *et al*., 2011). In most plants, RLK intracellular domain displays serine/threonine kinase (STK) specificity, while some of special receptors are receptor tyrosine kinases (Shiu and Bleecker, 2001; Xu *et al*., 2011). The temporal expression of RLKs during pollen development starts from microsporogenesis to pollen maturity, and the expression level varies across pollen development stages (Boisson‐Dernier *et al*., 2009; Skirpan *et al*., 2001). RLKs are also involved in pollen germination, growth and rupture of pollen tubes as well (Salem *et al*., 2011; Xu and Huang, 2014).

RLCK is a specific RLK family that it has no extracellular signal peptide domain and transmembrane domain. In this study, we report that ZmSTK1 and ZmSTK2, the most close pollen‐expressed homologs belonging to RLCK, are localized in the cytoplasm and regulate maize pollen development with additive effect. Further studies show that ZmSTKs interact with enolases and indirectly participate in the glycolysis pathway

## Results

### *ZmSTK1* and *ZmSTK2* have an additive effect on male gametophyte transmission

**Figure 1:**
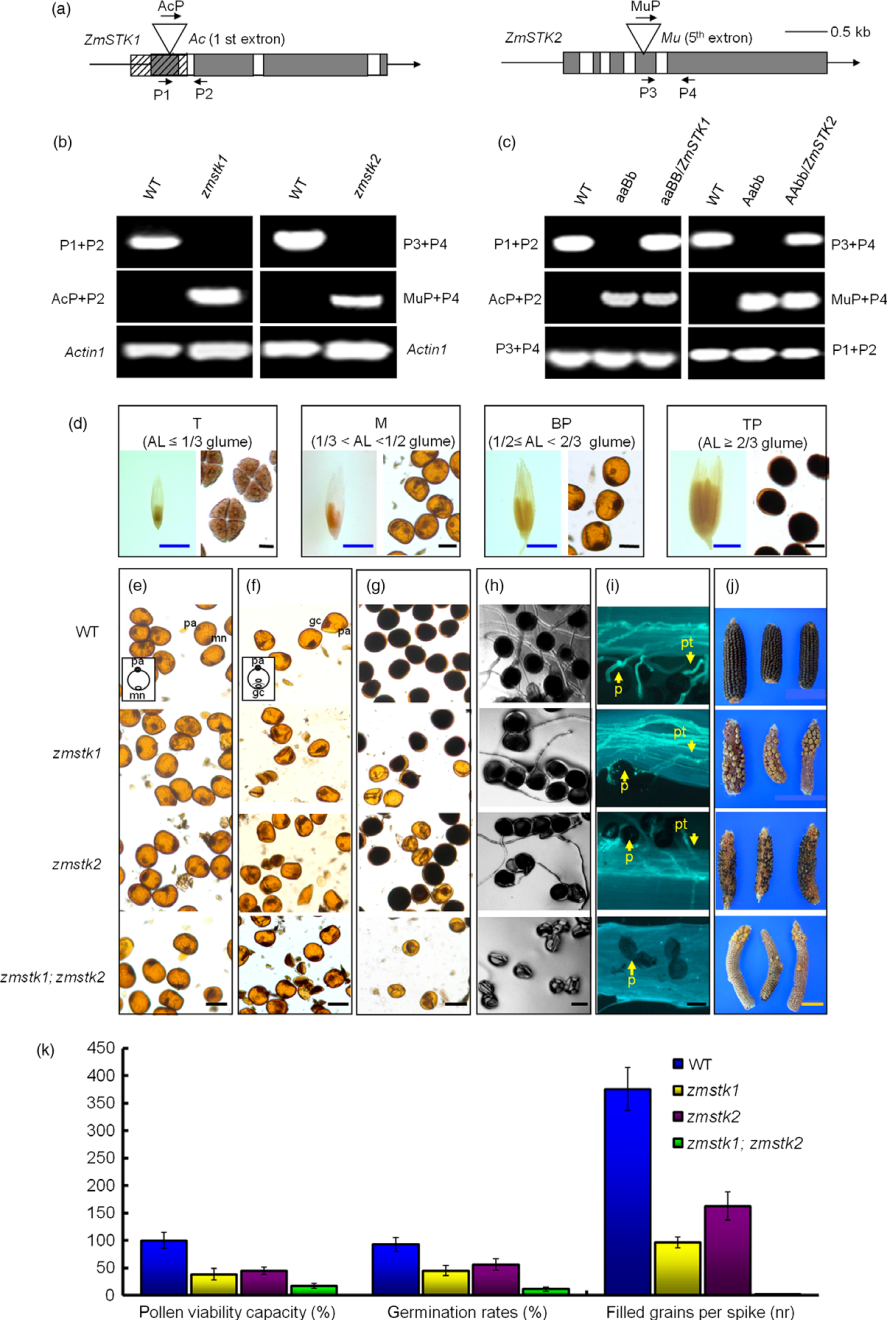
Characterization of *zmstk1* homozygous, *zmstk2* homozygous and double homozygous mutants. (a) Schematic diagrams for the *ZmSTK1* and *ZmSTK2* gene structures, and position of *Ac* or *Mu* insertions within the genes. White boxes and black boxes represent intron and exons, respectively. (b) Genotyping PCR of *zmstk1* and *zmstk2* mutants, *Actin1* was used as an internal control. (c) Identification of aaBb and Aabb mutant plants carrying the complementary transgenes. A denotes *ZmSTK1*; a denotes *zmstk1*; B denotes *ZmSTK2*; b denotes *zmstk2*. (d) The relationship between anther length and pollen developmental stages (AL denotes anther length; T denotes tetrad; M denotes microspore; BP denotes bicellular pollen; TP denotes tricellular pollen). (e) Microspore. (f) bicellular pollen. (g) tricellular pollen. (h) In vitro‐germinated pollen grains in medium at 8 h. (i) In vivo‐germinated pollen grains in maize filaments with aniline blue stain at 12 h. (j) Corn cobs. (k) Differences in pollen viability capacity (%), germination rates (%) and filled grains per spike (nr) were observed. Each error bar represents the mean of three independent experiments (±SE;* n* > 30 pollen grains from each genotype within visual field). Tetrad, microspore, bicellular pollen and tricellular pollen were stained with KI. Mn denotes microspore nuclei; pa denotes pollen apertures; gc denotes generative cells; p denotes pollen; pt denotes pollen tube. Black bars = 50 μm; yellow bars = 5 cm; blue bars = 5 mm. *ZmSTK2* data, including I_2_‐KI solution, pollen viability capacity (%), germination rates (%) and filled grains per spike (nr), refer to Javeed *et al*. (2017).

ZmSTK1 and ZmSTK2, two closest RLCK homologs in *Zea mays*, share 86% identity at the amino acid level (Wang *et al*., 2014; Zhou *et al*., 2014). We obtained *Ac*‐induced *zmstk1* mutant and *Mu*‐induced *zmstk2* mutant from Rutgers University, USA. *zmstk1* mutant has a 1.8‐kb *Ac* adjacent deletion that includes *ZmSTK1* promoters and extends into the second exon of *ZmSTK1* (Figure 1a) (Huang *et al*., 2017). *zmstk2* has a nonautonomous 1.4‐kb *Mu* element at the beginning of the 5th exon of *ZmSTK2* (Figure 1a) (Huang *et al*., 2017). In the field, both *zmstk1* and *zmstk2* maintain normal vegetative growth with ears developing similarly to those of the wild type but affect the number of kernels in an ear, which *zmstk1* has a strong kernel reduction, up to 40%, while *zmstk2* reduces 20% of those of the wild type.

To clarify the male‐specific functions of *ZmSTK1* and *ZmSTK2*, we performed reciprocal crosses between wild‐type plants and mutant lines. When wild type was male, we could not see any changes of kernel number in an ear of progenies compared to wild type. Once again, the progenies from the crosses Aabb (female) × WT or aaBb (female) × WT segregated 1:1 ratio with 50% double mutant alleles when genotyped by PCR (Table 2). These results demonstrated that *zmstk1* and *zmstk2* mutants were specific to the male gametophytes. Moreover, when Aabb or aaBb plants were used as pollen donors, the cotransmission of *zmstk1* and *zmstk2* was severely reduced (Table 2). However, when AaBB or AABb as a male parent, the transmission of *zmstk1* was lower than that of *zmstk2* (Table 2). These results demonstrated that, although female transmission was not affected, *ZmSTK1* and *ZmSTK2* genes had an additive and independent effect on male transmission.

To confirm that reduced male transmission was caused by the loss‐of‐function of Z*mSTK1* or *ZmSTK2*, complementation constructs *ZmSTK1* and *ZmSTK2* driven by the *CaMV 35S* promoter were transformed into Aabb and aaBb plants, respectively, via *Agrobacterium* LBA4404 transformation. The transgenic T_1_ kernels were selected on germination medium with hygromycin, and *ZmSTK1* or *ZmSTK2* coding sequence of the transgenic mutants was identified via PCR (Figure 1b) and Southern blotting (Figure S1). The male transmission of the transgenic mutants aaBb/*ZmSTK1* or Aabb/*ZmSTK2* was recovered by the introduction of each *ZmSTK* gene (Figure 1c), suggesting that disrupted *ZmSTK1* or *ZmSTK2* caused the failure of male transmission.

### *ZmSTK1* or *ZmSTK2* mutation alters male gametophyte development

The genetic analysis described above impelled us to examine pollen grains in transverse sections and starch accumulation in pollen grains during development. Based on anther length and microscopic observation (Figure 1d), pollen developmental stages were classified into tetrad stage (anther length ≤1/3 glume), microspore stage (1/3 glume < anther length < 1/2 glume), bicellular stage (1/2 glume ≤ anther length< 2/3 glume) and tricellular stage (anther length ≥ 2/3 glume) (Gagliardi *et al*., 1995).

In our experiment, starch accumulation in pollen grains started at bicellular stage (Figure 1f). As shown in Figure 1, almost all wild‐type pollen grains were circular (Figure 1e–g) and full of starch granules at tricellular stage visualized by I_2_‐KI solution (Figure 1g). Some of the bicellular pollen grains in aa or bb mutants started to collapse after microspore mitosis, accompanied with poor starch accumulation (Figure 1f). Almost all pollen grains of double mutants were collapsed, and starch accumulation was rarely observed (Figure 1g). Comparing with microspore and bicellular stages, the aberrant pollen grains in aa, bb and aabb mutants were conspicuous at tricellular stage (Figure 1g). Starch synthesized in pollen grains was apparently degraded during double mutant‐pollen development (Figure 1e–g). Abnormal pollen grains, containing no starch, were collapsed and smaller in size (approximately 40 μm in diameter) comparing with wild‐type pollen grains (approximately 50μm in diameter) (Figure 1e–g).

To obtain additional insights into aa, bb and aabb mutants phenotypes, a comparison of pollen viability capacity (%), germination rates (%) and numbers of filled grains per ear (number, nr) was performed. The pollen tubes of aa or bb mutants *in vivo* and *in vitro* grew lower than those of wild‐type plants, while aabb mutants almost had no germination (Figure 1h and i). Therefore, double homozygous mutants (aabb) had lowest pollen viability capacity (%), germination rates (%) and numbers of filled grains per ear (nr) than other genotypes (Figure 1k). Hence, under optimal growth conditions, *zmstk1* and *zmstk2* homozygous mutations result in significant impairment of pollen fitness and competitiveness both *in vivo* and *in vitro*, demonstrated that the deletion mutants carried null *zmstk1* or *zmstk2*, and consequently exhibited more severe pollen transmission deficiency phenotypes.

### Both *ZmSTK1* and *ZmSTK2* are expressed during late stages of pollen development, localized in the cytoplasm

**Figure 2:**
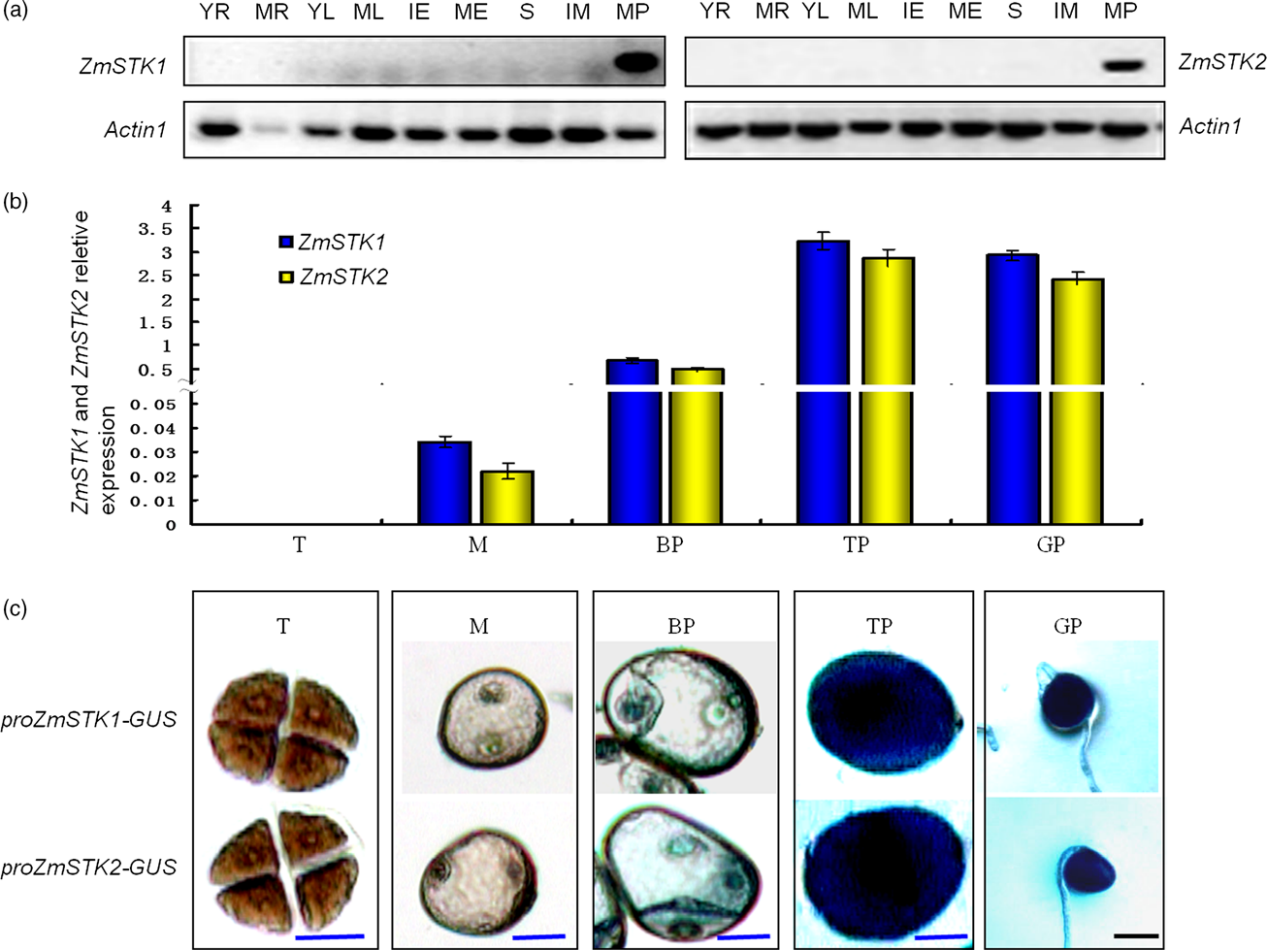
Spatial and temporal expression analyses of *ZmSTK1* and *ZmSTK2* in maize. (a) Evaluation of *ZmSTK1* and *ZmSTK2* expression pattern using northern blot analysis (actin mRNA was used as positive internal control. YR denotes young root; MR denotes mature root; YL denotes young leaf; ML denotes mature leaf; IE denotes immature embryos; ME denotes mature embryos; S denotes silks; IM denotes immature male flower; MP denotes mature pollen). (b) Expression patterns of *ZmSTK1* and *ZmSTK2* using qRT‐PCR (mean ± SD,* n* = 3). (c) Histochemical analysis of GUS expression in different pollen development stages of the transgenic plants. T denotes tetrad; M denotes microspore; BP denotes bicellular pollen; TP denotes tricellular pollen; GP denotes germinated pollen. Blue bars = 20 μm; black bars = 50 μm.

**Figure 3:**
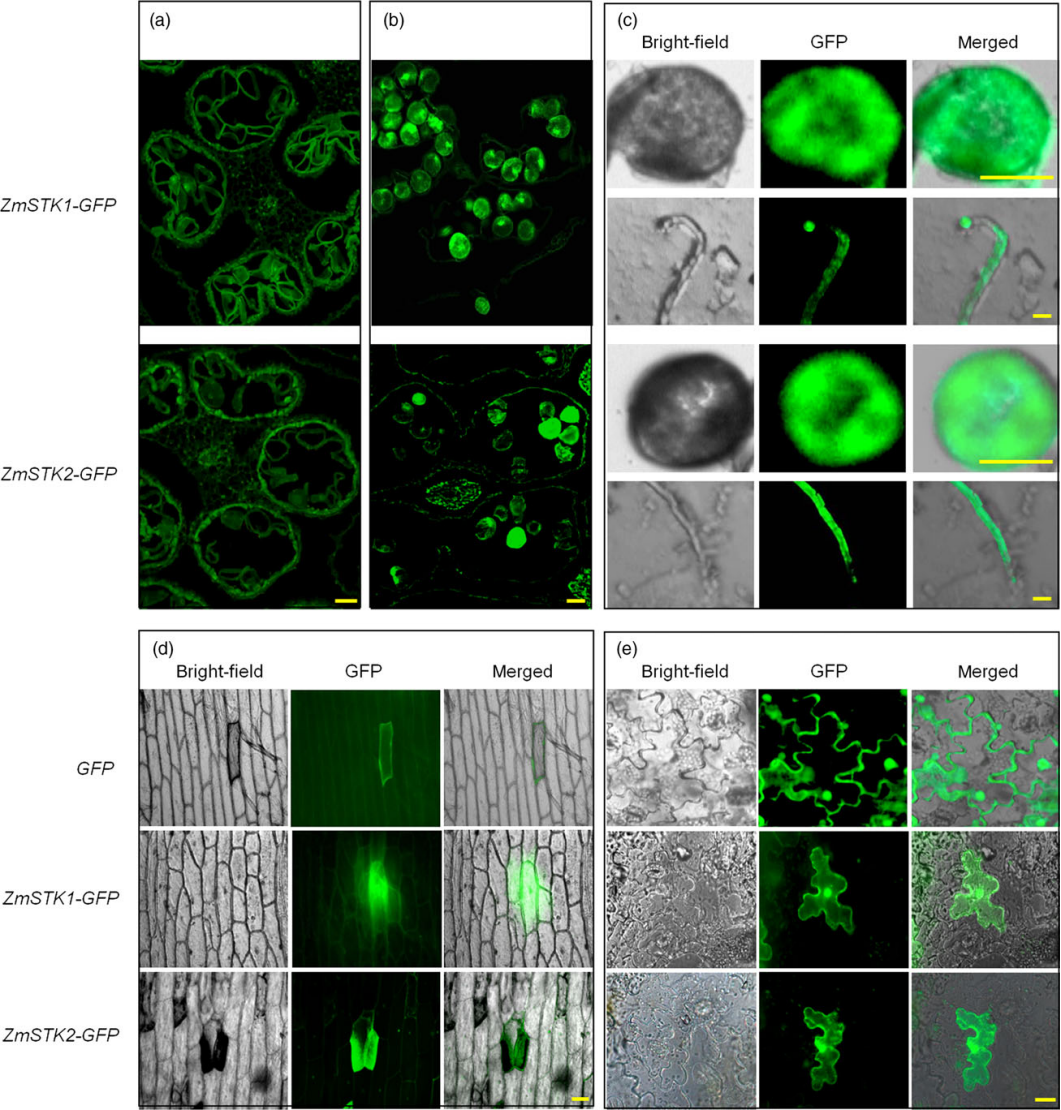
Localization of ZmSTK1 and ZmSTK2 in pollen, pollen tube and cells. (a) Fluorescence of *ZmSTK1‐GFP* and *ZmSTK2‐GFP* fusion proteins in bicellular pollen grains. (b) Fluorescence of *ZmSTK1‐GFP* and *ZmSTK2‐GFP* fusion proteins in tricellular pollen grains. (c) Fluorescence of *ZmSTK1‐GFP* and *ZmSTK2‐GFP* fusion proteins in germinated pollen grains and pollen tube. (d) Fluorescence of *ZmSTK1‐GFP* and *ZmSTK2‐GFP* fusion proteins in an onion epidermal cell. (e) Fluorescence of *ZmSTK1‐GFP* and *ZmSTK2‐GFP* fusion proteins in tobacco mesophyll cells. The *CaMV* *35S‐GFP* serves as control; bars = 50 μm.

To elucidate the functions of *ZmSTK1* and *ZmSTK2*, we investigated *ZmSTK1* and *ZmSTK2* expression in various maize tissues. The Maize eFP Browser expression database was employed to extract information about *ZmSTK1* and *ZmSTK2* expression. Both *ZmSTK1* and *ZmSTK2* are preferentially expressed in tassel and anther, and at especially high levels in anther (Figure S2). Northern blot results showed that *ZmSTK1* and *ZmSTK2* were expressed only in mature pollen and not expressed at all in other tissues (Figure 2a). Quantitative RT‐PCR analysis was used further to quantitatively characterize the expression level of these two genes in different stages of pollen development. Both *ZmSTK1* and ZmSTK2 hardly expressed at tetrad and microspore stage, started at bicellular stage, showed the peak at tricellular stage (Figure 2b). The GUS signals in the transgenic lines from genes *ZmSTK1* and *ZmSTK2* were observed in a similar pattern to those obtained by qRT‐PCR (Figure 2c). Further, we investigated the expression of *ZmSTK1* and *ZmSTK2* in male gametophyte using GFP fusion proteins. As expected, fluorescent signals of *ZmSTK1‐GFP* and *ZmSTK2‐GFP* were detected in mature pollen, but not in immature pollen (Figure 3a and b). Both *ZmSTK1‐GFP* and *ZmSTK2‐GFP* were observed to accumulate unevenly at the tip of the pollen tube (Figure 3c). These observations indicated that *ZmSTK1* and *ZmSTK2* expression occurred at later stages of pollen development and thus influenced pollen fertility.

In our previous study, ZmSTK1 and ZmSTK2 are grouped into RLCK, containing a universal stress protein (Usp) domain on N‐terminal, and a kinase domain on C‐terminal, but no transmembrane domain (Wang *et al*., 2014; Zhou *et al*., 2014). Therefore, we assume that both ZmSTK1 and ZmSTK2 are localized in the cytoplasm. To verify the subcellular localization of ZmSTK1 and ZmSTK2, fusion protein ZmSTK1‐GFP or ZmSTK2‐GFP was transiently expressed in onion epidermal cells and tobacco mesophyll cells (Figure 3d and e). Both ZmSTK1‐GFP and ZmSTK2‐GFP fluorescent signals were detected to uniformly distribute in the cytoplasm of tobacco mesophyll cells in 4‐week‐old tobacco seedlings, the same as in onion epidermal cells, indicating that ZmSTK1 and ZmSTK2 were cytoplasmic proteins, consistent with previous bioinformatic analysis (Wang *et al*., 2014; Zhou *et al*., 2014).

### ZmSTK1 or ZmSTK2 is present in different 55‐kDa complexes and interacts with the corresponding enolase1 or enolase2 protein at mature pollen stages

**Figure 4:**
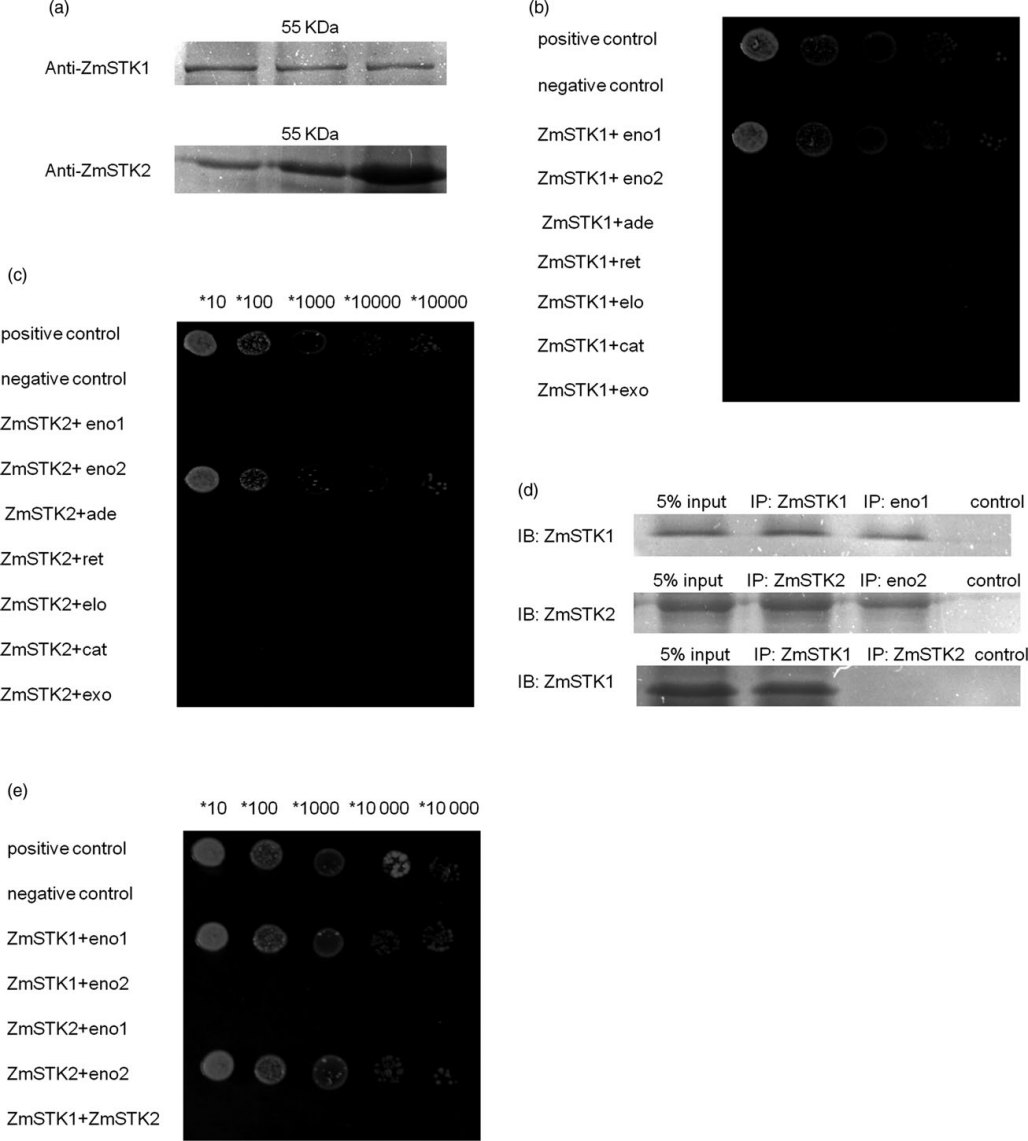
The proteins ZmSTK1 and ZmSTK2 interact with the enolase1 and enolase2 both in vitro and in vivo. (a) ZmSTK1 and ZmSTK2 protein complexes in maize mature pollen. (b and c) Yeast two‐hybrid analysis of ZmSTK1 and enolase1, ZmSTK2 and enolase2 (positive denotes cotransformation with the positive plasmids pGBKT7‐53p and GADT7‐RecT; negative denotes cotransformation with the negative plasmids pGBKT7‐Lam and GADT7‐RecT). (d) The Co‐IP results using serum against ZmSTK1 or enolase1, ZmSTK2 or enolase2, and ZmSTK1 or ZmSTK2. The negative controls are the antibodies against ZmSTK1 or enolase1, ZmSTK2 or enolase2, and ZmSTK1 or ZmSTK2 in RIPA buffer in the absence of the crude protein extract. (e) Yeast two‐hybrid analysis of various ZmSTK1, enolase1, ZmSTK2 and enolase2 constructs. eno1 denotes enolase1 (GRMZM2G064302); eno2 denotes enolase2 (GRMZM2G048371); ade denotes adenosylhomocysteinase (GRMZM2G111909); ret denotes reticuline oxidase precursor (GRMZM2G080907); elo denotes elongation factor 1‐alpha (GRMZM2G153541); cat denotes catalase isozyme B (GRMZM2G088212); exo denotes exopolygalacturonase (GRMZM2G418644).

To examine the interaction proteins with ZmSTK1 or ZmSTK2 at mature pollen stages, the crude protein extracts from mature pollen grains were isolated and the Co‐IP was first performed. A 55‐kDa protein complex was identified via Western blot using anti‐ZmSTK1 or anti‐ZmSTK2 serums, respectively (Figure 4a). Together, a total of seven proteins as the components of these 55‐kDa protein complexes were identified, including enolase1 (NP_001105896), enolase2 (NP_001105371), adenosylhomocysteinase (NP_001148534), reticuline oxidase precursor (NP_001148634), elongation factor 1‐alpha (NP_001151074), catalase isozyme B (NP_001241808) and exopolygalacturonase (AFW80846). Second, Y2H was employed to confirm the interaction proteins among the seven proteins with ZmSTK1 or ZmSTK2. The results indicated that ZmSTK1 interacted with enolase1, and ZmSTK2 interacted with enolase2, there were no more interactions detected between ZmSTK1 and ZmSTK2, and each of ZmSTKs with the other five proteins (Figure 4b, c and e). Once again, Co‐IP was used to validate the ZmSTK1‐enolase1 or ZmSTK2‐enolase2 interaction at the mature pollen stages. Anti‐ZmSTK1 co‐immunoprecipitated with enolase 1 but not with ZmSTK2, and anti‐ZmSTK2 co‐immunoprecipitated with enolase2 (Figure 4d). These results demonstrated that enolase1 was in the ZmSTK1 complex and enolase2 in the ZmSTK2 complex in maize mature pollen grains, indicating that ZmSTK1 and ZmSTK2 regulated the maize pollen development through similar but different pathways.

**Figure 5:**
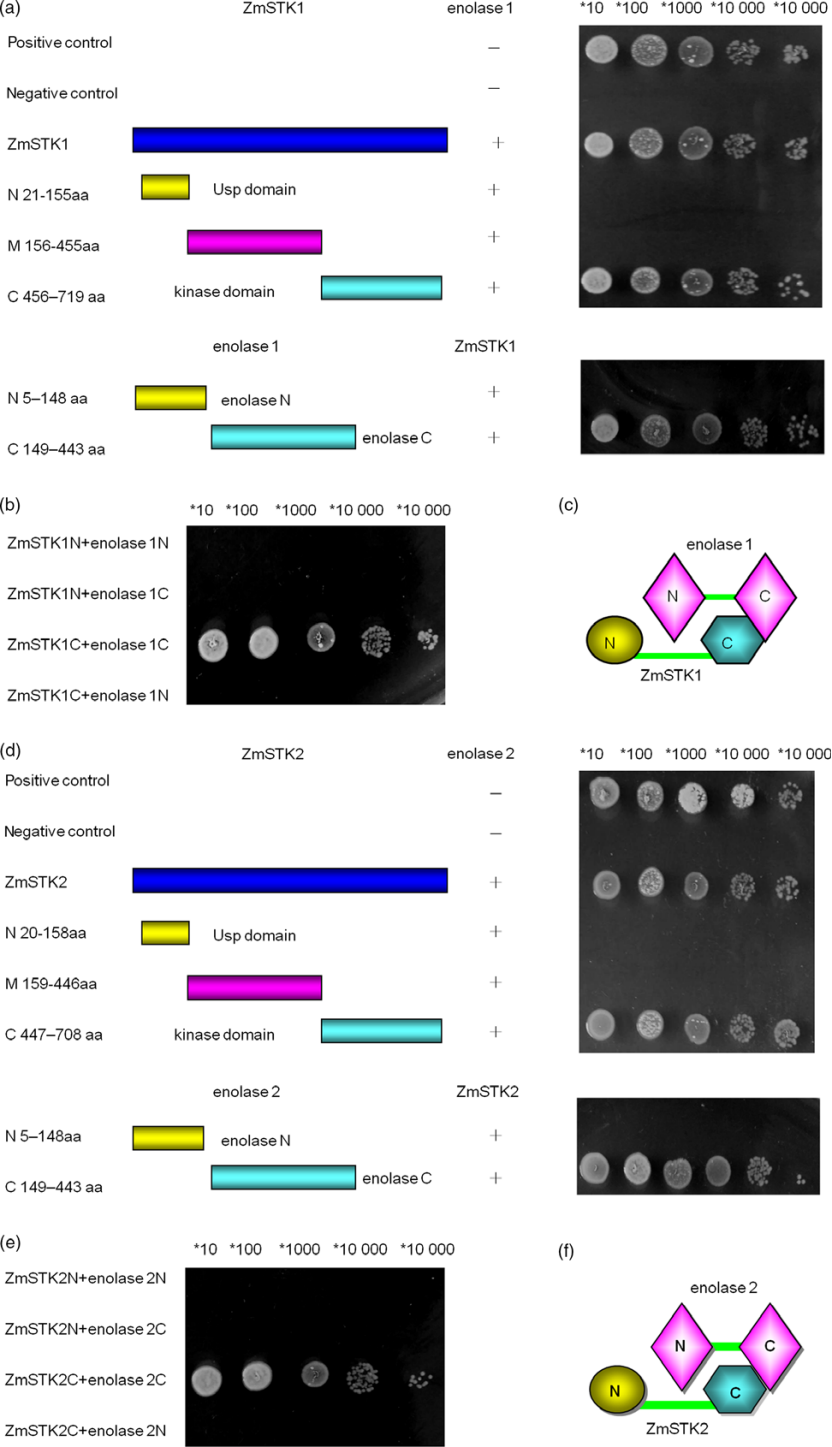
The patterns of interactions between proteins ZmSTKs and enolases. (a and b) Yeast two‐hybrid analysis of various ZmSTK1 and enolase1 constructs (positive denotes cotransformation with the positive plasmids pGBKT7‐53p and GADT7‐RecT; negative denotes cotransformation with the negative plasmids pGBKT7‐Lam and GADT7‐RecT). (c) An interaction model for proteins ZmSTK1 and enolase1. (d and e) Yeast two‐hybrid analysis of various ZmSTK2 and enolase2 constructs (positive denotes cotransformation with the positive plasmids pGBKT7‐53p and GADT7‐RecT; negative denotes cotransformation with the negative plasmids pGBKT7‐Lam and GADT7‐RecT). (f) An interaction model for proteins ZmSTK2 and enolase2.

To further characterize which domains were responsible for ZmSTK1‐enolase1 or ZmSTK2‐enolase2 interaction, N‐terminal, C‐terminal and middle fragment between N‐terminal and C‐terminal of ZmSTK1, ZmSTK2, enolase1 and enolase2 by inserting random deletion mutations were cloned into vectors. Y2H assays were used to discover interactions among reciprocal hybrids of these truncated fragments. As shown in Figure 5a–f, kinase domain (in C‐terminal, 456–719aa) of ZmSTK1 interacted with C‐terminus (149–443aa) of enolase1, and ZmSTK2‐enolase2 shared the same interaction pattern as that of ZmSTK1‐enolase1.

**Figure 6:**
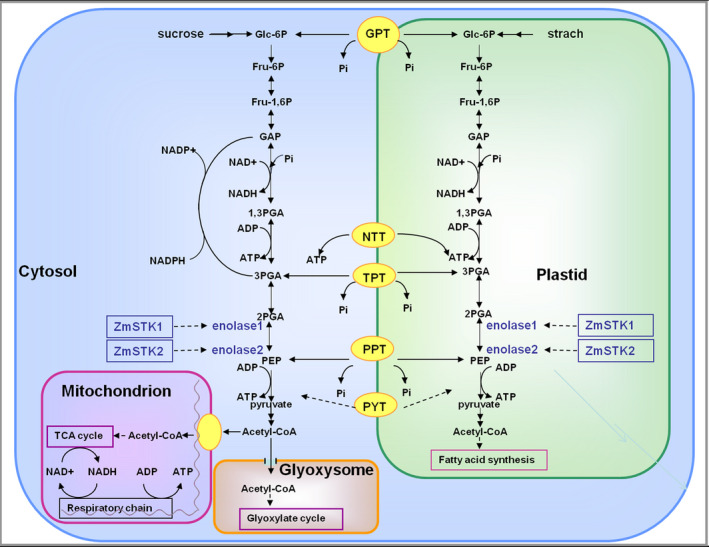
Proposed model for metabolic pathways interacting with ZmSTK1 and ZmSTK2 in mature pollen grains. PYT denotes pyruvate translocator; GPT denotes glucose phosphate/Pi translocator; PPT denotes phosphoenolpyruvate/Pi translocator; TPT denotes triose phosphate/Pi translocator; Fru‐1, 6P denotes fructose 1,6 bisphosphate; Fru‐6P denotes fructose 6 phosphate; GAP denotes glyceraldehyde‐3‐phosphate; Glc‐6P denotes glucose 6 phosphate; PEP denotes phosphoenolpyruvate; 1,3‐PGA denotes 1,3‐bisphosphoglycerate; 2‐PGA denotes 2‐phosphoglycerate; 3‐PGA denotes 3‐phosphoglycerate.

Using SWISS‐MODEL (https://swissmodel.expasy.org/), the structure models of ZmSTKs‐KD (kinase domain) and C‐terminus of enolases were constructed (Figure S3). Further, we used software KinasePhos (http://kinasephos.mbc.nctu.edu.tw/) and MotifScan (https://myhits.isb-sib.ch/cgi-bin/motif_scan) to predict protein binding sites and kinase phosphorylation sites of ZmSTKs‐KD, and enolases’ C‐terminuses (Figure S3). The results showed that the three‐dimensional structures between ZmSTK1‐KD and ZmSTK2‐KD were not significantly different. However, protein binding sites and kinase phosphorylation sites of ZmSTKs‐KD and C‐terminus of enolases showed significant differences. Enolase is a key enzyme in glycolytic pathway (Armand *et al*., 2016; Jennifer and Renate, 2014). Thus, ZmSTKs interact with enolases and indirectly participate in the glycolysis pathway (Figure 6).

### *ZmSTK1* or *ZmSTK2* mutation decreases the activity of enolase1 or 2 and contents of metabolic substrates in mature pollen grains

**Figure 7:**
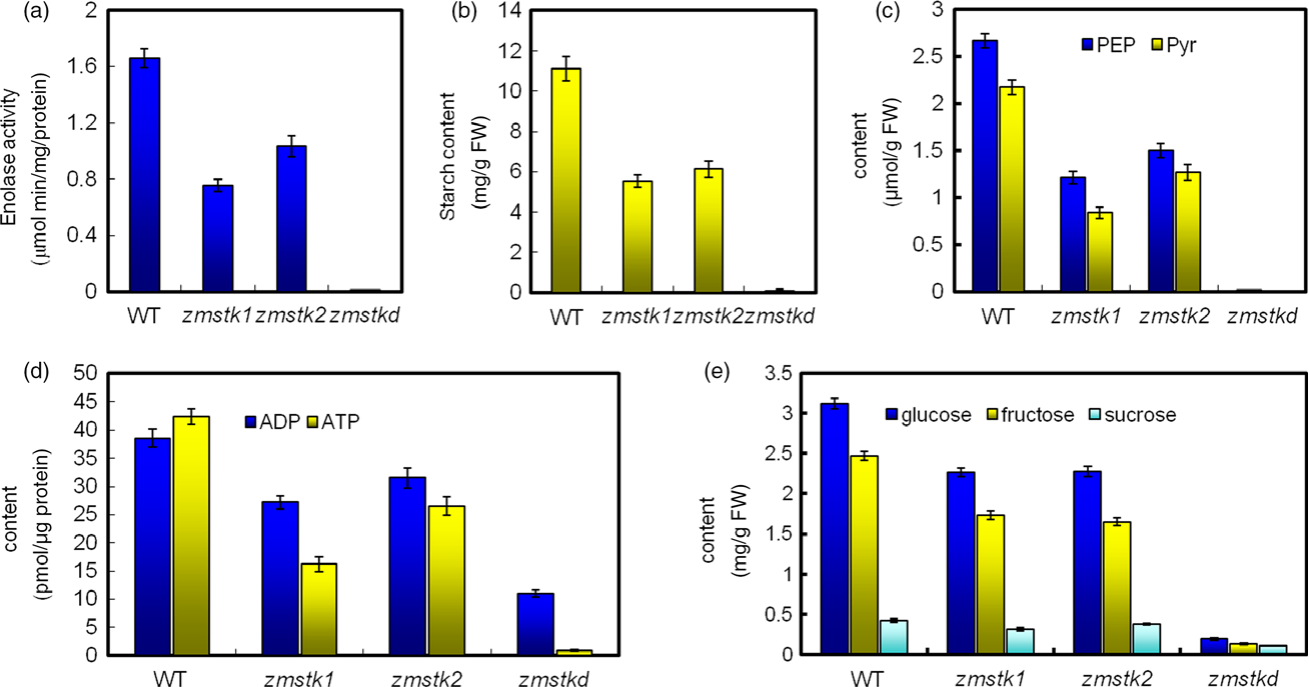
Activity of enolases and contents of metabolites in the mature pollen grains. (a) Activities of enolases. (b) Content of starch. (c) Contents of PEP and pyruvate. (d) Contents of ADP and ATP. (e) Contents of glucose, sucrose and fructose. Values are mean ± SE (*n* = 3).

Upon detecting the metabolic substrates of glycolysis pathway in the mutants, mature pollen grains were subjected to the activity of enolases and contents of PEP, pyruvate, ADP/ATP, starch, glucose, sucrose and fructose. Compared with wide type in enolase activity, *zmstk1* mutants (aa) were remarkably decreased by 54.50%, *zmstk2* mutants (bb) by 37.78% while double mutants (aabb) by 99.46% (Figure 7a).

For the contents of metabolic substrates in mature pollen grains comparing with wild type, *zmstk1* mutants reduced 1.33 μmol/g PEP, 1.45 μmol/g pyruvate, 11.33 pmol/μg ADP, 26.15 pmol/μg ATP, 5.56 mg/g starch, 0.86 mg/g glucose, 0.11 mg/g sucrose and 0.84 mg/g fructose; *zmstk2* mutants reduced 0.90 μmol/g PEP, 1.16 μmol/g pyruvate, 7.04 pmol/μg ADP, 15.86 pmol/μg ATP, 4.98 mg/g starch, 0.74 mg/g glucose, 0.04 mg/g sucrose and 0.82 mg/g fructose; and the double mutants showed the strongest reduction in the contents of PEP, pyruvate, ADP/ATP, starch, glucose, sucrose and fructose (Figure 7b–e).

To further clarify the male‐specific functions of ZmSTK1 and ZmSTK2, we checked the activity of enolases and contents of PEP, pyruvate, ADP/ATP, starch, glucose, sucrose and fructose in immature ears. The results showed no significant differences between wild type and mutant immature ears (Figure S4).

## Discussion

Prior study revealed that ZmSTK1 and ZmSTK2 are two closest RLCK homologs in *Zea mays* as members of RLK subfamily, sharing 86% identity at the amino acid level, in which contains an Usp receptor domain and lacks an extracellular domain (Wang *et al*., 2014; Zhou *et al*., 2014). Subcellular localization confirmed that ZmSTK1 and ZmSTK2 were cytosolic proteins. The disruption of *ZmSTK1* or *ZmSTK2* reduced the number of kernels in an ear up to 40% and 20% of those of the wild type, respectively (Huang *et al*., 2017). Many studies demonstrated that the same gene family members regulated pollen germination and pollen tube growth in the form of functional redundancy or functional differences (Boisson‐Dernier *et al*., 2013; Hord *et al*., 2008; Liu *et al*., 2013; Miyazaki *et al*., 2009). However, our genetic analysis showed that *ZmSTK1* and *ZmSTK2* acted additively to alter male gametophyte development. Using *Ac*‐ and *Mu*‐insertion mutants, the male‐specific functions of *ZmSTK1* and *ZmSTK2* were clarified. The *ZmSTK1* and *ZmSTK2* mutation showed a stronger pollen collapse and reduced male gametophyte transmission phenotypes comparing with wild types (Figure 1; Tables 1 and 2), confirming that the *zmstk1* and *zmstk2* mutants were adverse factors that were previously observed in the deletion and insertion alleles (Huang *et al*., 2017). During maize pollen development, late microspores with *zmstk1*,* zmstk2* and double homozygous mutation gave rise to normal vegetative and generative cells, but abnormalities were evident at bicellular pollen stage. The number of abnormal pollen grains of double homozygous mutation increased at bicellular pollen stage, and almost all pollen grains were collapsed at tricellular stage (Figure 1). In addition, transcripts of *ZmSTK1* and *ZmSTK2* were preferentially accumulated at the tricellular pollen grains (Figure 2). These observations indicated that *ZmSTK1* and *ZmSTK2* expression occurred during the later stages of pollen development, and influenced pollen fertility.

**Table 1:** Complementation analysis of the zmstk1 and zmstk2 double mutant

Parental Genotypes (Female × Male)	Genotype of progeny									Expected	Observed
	A_B_				A_bb		aaB_				
	AABB	AABb	AaBB	AaBb	AAbb	Aabb	aaBB	aaBb	aabb		
AaBb × AaBb	29	34	186	77	54	46	49	42	28		
Total		285				191			28	9:6:1	10.18:6.82:1

**Table 2:** Transmission analysis of *zmstk1* and *zmstk2* mutant alleles

Female	Male	Progeny		Total	Expected	Observed
Cross						
WT	AaBB	104 (AaBB)	154(AABB)	258	1:1	0.68: 1
AaBB	WT	197(AaBB)	186(AABB)	383	1:1	1.06:1
WT	AABb	129(AABb)	159(AABB)	288	1:1	0.81:1
AABb	WT	189(AABb)	175(AABB)	364	1:1	1.08:1
WT	aaBb	5(AaBb)	54 (AaBB)	58	1:1	0.09:1^a^
aaBb	WT	154(AaBb)	166(AaBB)	320	1:1	0.93: 1
WT	Aabb	7(AaBb)	69(AABb)	75	1:1	0.10:1^a^
Aabb	WT	164(AaBb)	152(AaBB)	316	1:1	1.08: 1

Parent genotype	Genotype of progeny			Expected	Observed
Complementation analysis					
aaBb/*ZmSTK1*	aaBB/*ZmSTK1*	aaBb/*ZmSTK1*	aabb/*ZmSTK1*	1:2:1	1:2.16:0.94
	31	67	29		
Aabb/*ZmSTK2*	AAbb/*ZmSTK2*	Aabb/*ZmSTK2*	aabb/*ZmSTK2*	1:2:1	1:1.93:0.89
	46	89	41		

a Significantly different from the expected 1:1 segregation ratio (*P* < 0.001).

Recent studies have revealed the signal model of RLKs in the regulation of pollen development. AtPRK2, a pollen‐specific RLK, acts as a positive regulator of the ROP1 (Rho‐like small GTPase from plant) signalling pathway most likely activated by RopGEF1 (Rho guanine nucleotide exchange factors) through phosphorylation (Chang *et al*., 2013). The juxtamembrane domain of pollen‐specific RLK LePRK2 requires its kinase domain for interaction with LePRK1, associating with RopGEF, to promote pollen tube polar growth within styles (Kaothien *et al*., 2005; Salem *et al*., 2011). In pollen tube, ANX1 and ANX2 might receive localized external cues, and the signal could be transferred to their direct binding partner RopGEFs which activate ROP GTPases (Kanaoka and Torii, 2010). Together, these studies suggest that RLKs are likely to work as an upstream regulator of ROP signalling via RopGEFs. In the present study, ZmSTKs cross‐interacted with enolases (Figures 4 and 5). Enolase2, located in chromosome 1S, shares 75.6% nucleotide and 89.5% deduced amino acid sequence identity with enolase1 located in chromosome 9S (Lal *et al*., 1998; Peschke and Sachs, 1994). With the help of bioinformatic analysis, we discovered that protein binding sites and kinase phosphorylation sites of ZmSTKs‐KD and C‐terminus of enolases showed significant differences. These results suggested that the different amino acid sites might contribute to the interaction differences between ZmSTK1‐KD and C‐terminus of enolase1, ZmSTK2‐KD and C‐terminus of enolase2. Our results add on to the complex interaction network surrounding pollen‐specific RLKs from maize and disclosed a variety of unexpected interaction possibilities (Figure 6).

Many studies show that the knocked‐out genes coding for glycolytic enzymes and related proteins specifically affect pollen development (Muñoz‐Bertomeu *et al*., 2010; Prabhakar *et al*., 2010). Enolase, as part of the glycolytic pathway, is a key enzyme in glycolysis and catalyses the reversible dehydration of 2‐phosphoglycerate (2‐PGA) to phosphoenolpyruvate (PEP) (Prabhakar *et al*., 2010). Further downstream PEP is catalysed to pyruvate, consuming ADP to synthesize ATP at the same time. Plants with defective enolase function showed severe growth defects, including defective anthesis, defective pollen tube elongation and defective female reproductive organ development, resulting in sterility (Eremina *et al*., 2015). Our results showed that plants with defective *ZmSTK1* and *ZmSTK2* function produced collapsed pollen grains and reduced the activity of enolases and contents of PEP, pyruvate, ADP/ATP.

Both PEP and pyruvate in cytoplasm could be used as source metabolites to introduce carbon into the plastid. The contents of starch and soluble sugar in pollen grains are very important for pollen fertility. The reason is that starch accumulated during late stages of pollen development is suggested to be an energy reserve for pollen tube germination and also provides a metabolic checkpoint of pollen maturity (Datta *et al*., 2001, 2002; Pring and Tang, 2004). Also, high levels of sucrose would preserve pollen viability (Guarnieri *et al*., 2006). Further compared with male‐fertile tissues, the levels of glucose and fructose showed significant reduction in male‐sterile during maize pollen development (Datta *et al*., 2002). In the present study, the contents of starch, glucose, sucrose and fructose in maize pollen grains of double mutants were significantly reduced. It might be due to the loss of ZmSTKs’ serine/threonine kinase activity after the mutations, resulting in the loss of the activity of enolase1 and enolase2, further the disorder of glycolysis metabolism. These results suggested that ZmSTK1 and ZmSTK2 control the activity of enolases and take part in glycolytic pathway. Thus, we proposed a model to explain the regulatory mechanism for *ZmSTK1* and *ZmSTK2* involved in maize pollen development with additive effect (Figure 6). These findings may be of reference meaning for the understanding of the male sterility mechanism in maize hybrid breeding.

## Experimental procedures

### Plant materials, growth conditions and genetic analysis

The primers used in this study were listed in Table S1. All the stocks used in this study are in the inbred McC background (Dooner and Nelson, 1977; Ralston *et al*., 1988). The *Ac*‐induced mutant *zmstk1* homozygous (*ZmSTK1*, grmzm2g165433) and *Mu*‐induced mutant *zmstk2* homozygous (*ZmSTK2*, grmzm2g301647) were previously published (Fu *et al*., 2001; Wang *et al*., 2014; Zhou *et al*., 2014). The inbred McC (control), mutants and transgenic lines were grown under greenhouse conditions with 16‐h light/8‐h dark cycle (diurnal temperature cycle gradually changes from 10 °C at 2:00 am to 25 °C at 2:00 pm, 150 μmol photons/m^2^/s, 50%–80% relative humidity).

For genetic analysis, z*mstk1* homozygous and *zmstk2* homozygous mutants were crossed to each other and double mutant combinations (F_2_) were genotyped by PCR using the specific primers listed in Table S1. The kernel number per ear was measured after harvest.

### *In vitro* pollen growth assays

Mature pollen grains of wild types and mutants were brushed onto slides containing germination medium described by Schreiber and Dresselhaus (2003). Slides were incubated in incubation boxes for 8 h at 25 °C. The germination rate was calculated after 8‐h incubation.

I_2_‐KI [0.5 g iodine (I_2_) + 1 g potassium iodide (KI)] and aniline blue staining (Park and Twell, 2001) were performed on glass slides. Stained pollen grains and pollen tubes were visualized and photographed using Olympus fluorescence microscope BX51TF.

### Generation of the complementation construct and *Zea mays* transformation

The coding sequences of *ZmSTK1* and *ZmSTK2* were amplified using McC cDNA with gene‐specific primers (Table S1). After verification by sequencing, the fragments were cloned into pCAMBIA1301. The *CaMV 35S‐ZmSTK1* and *CaMV 35S‐ZmSTK2* plasmids were separately transformed into *zmstk1* and *zmstk2* mutants by *Agrobacterium* LBA4404 transformation as described by Wang *et al*. (2007).

### Southern blot analysis

Leaf DNA from wild‐type McC (negative control), *CaMV 35S‐ZmSTK1* and *CaMV 35S‐ZmSTK2* transgenic plants was isolated. Restriction digested DNA (10 μg) was resolved on 0.8% agarose gels and transferred to nylon membranes. The DNA‐fixed membranes were hybridized with the digoxigenin (DIG)‐labelled Cym probes and exposed to X‐ray film for autoradiography according to the manufacturer's manual for the DIG High Prime DNA Labeling and Detection Starter Kit I (Roche, Shanghai, China).

### RNA isolation, northern blot analysis and quantitative RT‐PCR

RNA was isolated from different tissues of maize inbred line McC according to the method described by Lu *et al*. (2013). For northern blot analysis, RNA samples were transferred onto nylon membranes and hybridized to random primer labelled P^32^ probes. Hybridization and washing conditions were performed according to a previous study (Sambrook *et al*., 1989). Quantitative RT‐PCR analysis was performed using the ABI7500 system (Bio‐Rad) with the SYBR Green qPCR Master Mix (TaKaRa), following the previous study (Lu *et al*., 2013). Z*ea mays actin1* was selected as the internal control.

### The subcellular localization of ZmSTK1 and ZmSTK2 in onion epidermal cells and tobacco mesophyll cells

The coding sequences of *ZmSTK1* and *ZmSTK2* were constructed into the *pCAMBIA1301‐GFP* vector in the C‐terminal. Empty vector and GFP fusion proteins were transiently expressed in onion epidermal cells as described previously (Shi *et al*., 2015) and Tobacco mesophyll cell described by Tamura *et al*. (2013). GFP fluorescence was analysed using Olympus fluorescence microscope BX51TF.

### Construction of *proZmSTK1‐GUS* and *proZmSTK2‐GUS* reporter gene cassettes and plant transformation

Promoters of *ZmSTK1* and *ZmSTK2* were used to initiate the expression of *GUS* reporter gene. *proZmSTK1,* a 1,127‐bp fragment of the upstream of putative transcription start site of *ZmSTK1,* and *proZmSTK2,* a 2,100‐bp fragment of upstream of putative transcription start site of *ZmSTK2,* were fused in‐frame to GUS reporter gene. The *proZmSTK1‐GUS* or *proZmSTK2‐GUS* vector was transferred into *Agrobacterium* LBA4404, and transgenic plants were generated as described by Wang *et al*. (2007).

### Co‐IP experiments

To explore the complexes combined with ZmSTK1 or ZmSTK2, the crude protein extracts from mature pollen grains were obtained and Co‐IP was performed according to the method described by Huh *et al*. (2015). To further verify whether ZmSTK1 and enolase1 or ZmSTK2 and enolase2 are expressed in the same complex, Co‐IP was carried out again using the above‐mentioned method. MS/MS analysis and Western blot analysis were performed as described previously (Sun *et al*., 2012).

### Yeast two‐hybrid analysis

To identify the interactions with enolase1 or enolase2 protein, the full‐length and various deletion constructs of genes *ZmSTK1* and *ZmSTK2* were cloned into pGBKT7 bait vector, respectively. The full‐length and various deletion constructs of genes *enolase1* and *enolase2* were cloned into pGADT7 prey vector, respectively.

Yeast strain AH109 was cotransformed with different plasmid combinations, and successful cotransformation was confirmed on SD‐leu/‐try, or SD‐leu/‐try/‐his, or SD‐leu/‐try/‐his/‐ade (Shanghai Genomics, Shanghai, China). Yeast strains cotransformed with pGBKT7‐p53 and pGADT7‐T or with pGBKT7‐lam and pGADT7‐T were used as positive control and negative control, respectively.

### The analysis of enolase activity and metabolite contents

Mature pollen grains and immature maize ears (female inflorescences) of McC, *zmstk1* homozygous (aa), *zmstk2* homozygous (bb) and double homozygous mutants (aabb) lines were collected. The extraction and analysis of glucose, sucrose and fructose were described in Kerr *et al*. (1985). Starch was measured as described in Dyson *et al*. (2015). The extraction and analysis of pyruvate and phosphoenolpyruvate (PEP) were described in Li *et al*. (2015). ADP and ATP were measured according to Rieder and Neuhaus (2011). The enolase activity was measured spectrophotometrically following the direction of PEP formation described by Prabhakar *et al*. (2009).

## Supporting information


**Figure S1** Identification and molecular characterization of *ZmSTK1* and *ZmSTK2* overexpression in the T_1_ generation of maize.
**Figure S2** Tissues and development‐specific expression data of *ZmSTK1* and *ZmSTK2* in maize.
**Figure S3** Structure models of ZmSTKs‐KD and C‐terminus of enolases.
**Figure S4** Activities of enolases and contents of metabolites in the immature ears.

**Table S1** The list of primers used in the study.
