# Supplementary material for: ZmSTK1 and ZmSTK2, encoding receptor‐like cytoplasmic kinase, are involved in maize pollen development with additive effect
Source: Plant Biotechnol J. 2018 Feb 13;16(8):1402–14. doi: 10.1111/pbi.12880 (PMC6041449; doi:10.1111/pbi.12880)
Supplement: Supplementary file 1 — Figure S1 Identification and molecular characterization of ZmSTK1 and ZmSTK2 overexpression in the T1 generation of maize. Figure S2 Tissues and development‐specific expression data of ZmSTK1 and ZmSTK2 in maize. Figure S3 Structure models of ZmSTKs‐KD and C‐terminus of enolases. Figure S4 Activities of enolases and contents of metabolites in the immature ears. [file PBI-16-1402-s002.pdf]

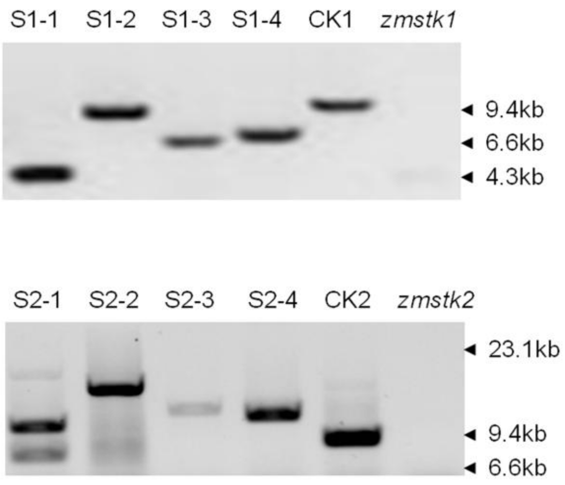

Figure S1 Identification and molecular characterization of *ZmSTK1* and *ZmSTK2* over-expression in the T_1 generation of maize. Genomic DNA was taken from typical individuals of transgenic maize lines digested completely with *Eco*R V before Southern analysis. DNA from untransformed plants was used as a negative control; vectors CK1 (pCAMBIA1301-*ZmSTK1*) and CK2 (pCAMBIA1301-*ZmSTK2*) was used as a positive control. *ZmSTK1* and *ZmSTK2* coding sequences were used as probes. *ZmSTK1* and *ZmSTK2* were inserted between the *CaMV 35S* promoter and the NOS terminator in pCAMBIA1301. S1 denotes *ZmSTK1* transgenic plants; S2 denotes *ZmSTK2* transgenic plants.

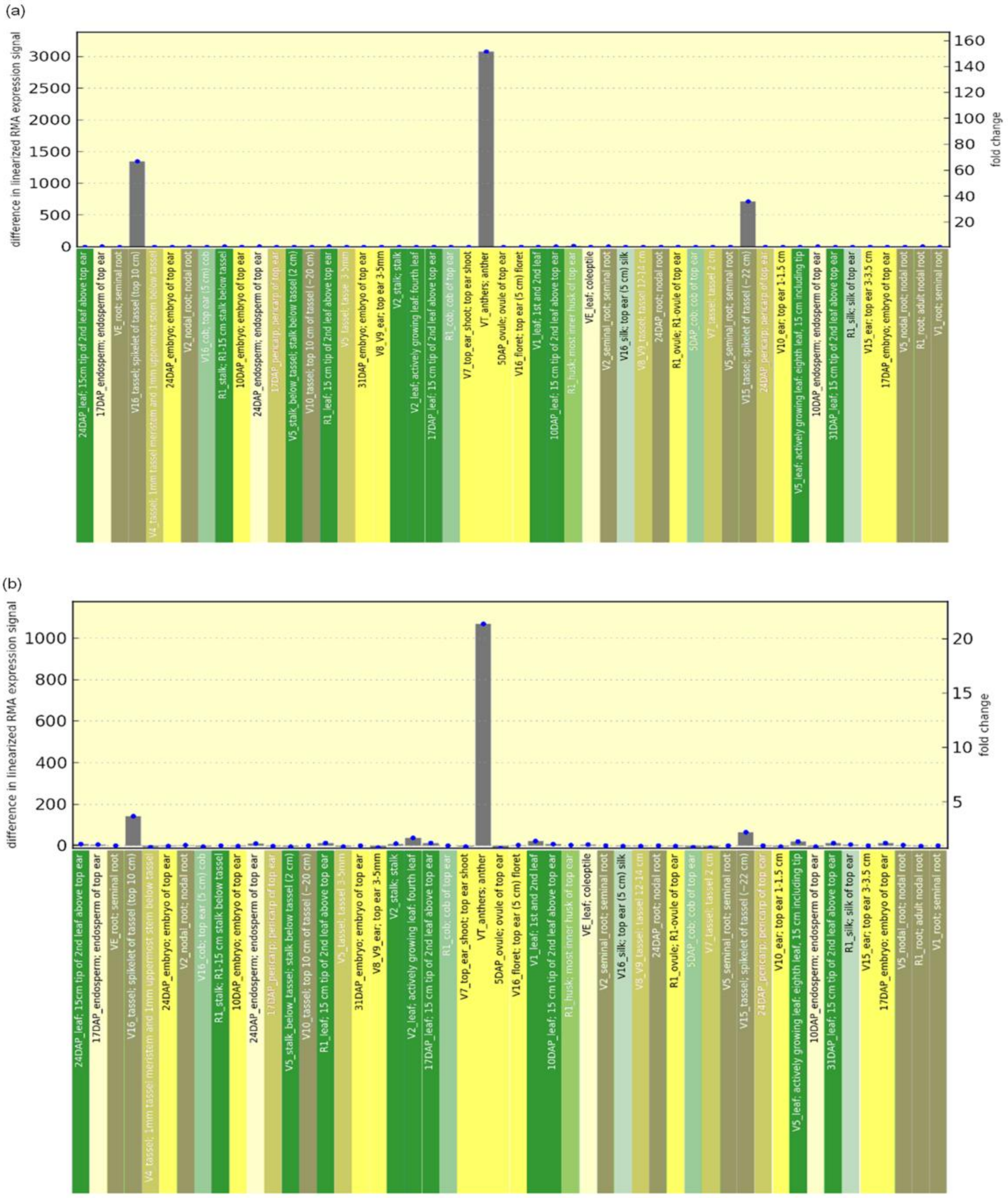

Figure S2 Tissues and development-specific expression data of *ZmSTK1* and *ZmSTK2* in maize. (a) Expression profiles of *ZmSTK1* (grmzm2G165433) were obtained from the Maize eFP Browser. (b) Expression profiles of *ZmSTK2* (grmzm2g301647) were obtained from the Maize eFP Browser.

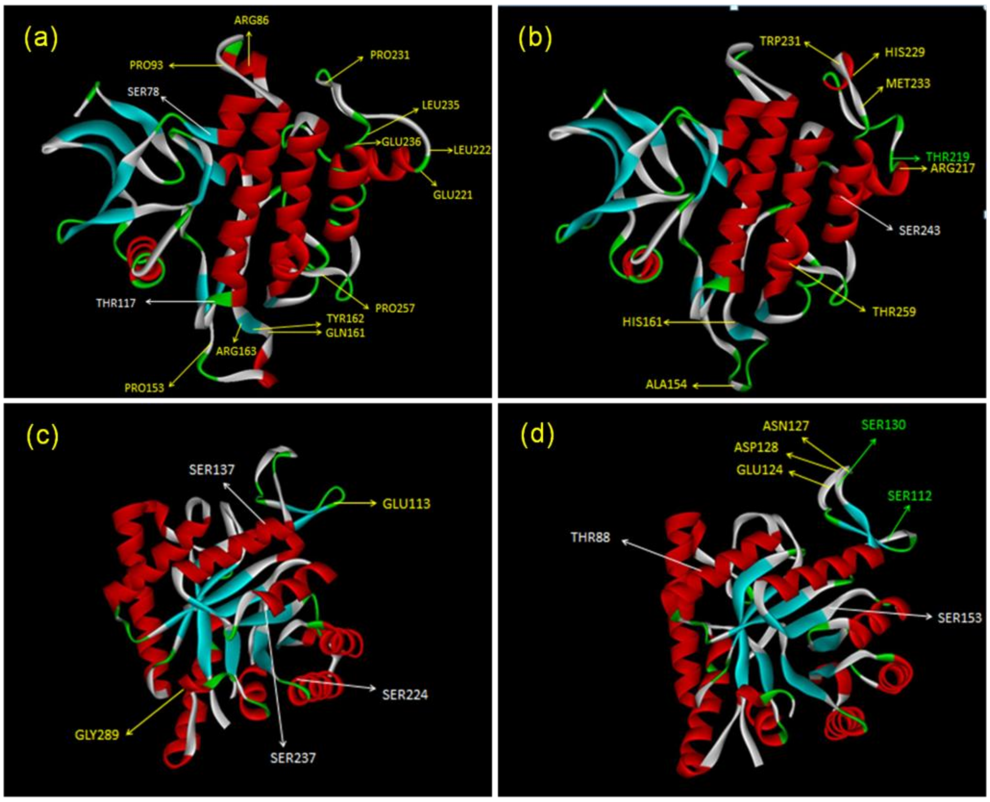

Figure S3 Structure models of ZmSTKs-KD and C-terminus of enolases. (a) The structure model of ZmSTK1-KD. (b) The structure model of ZmSTK2-KD. (c) The structure model of C-terminus of enolase1. (d) The structure model of C-terminus of enolase2. White arrows denote different kinase phosphorylation sites. Yellow arrows donate different protein binding sites. Green arrows denote both different kinase phosphorylation sites and different protein binding sites.

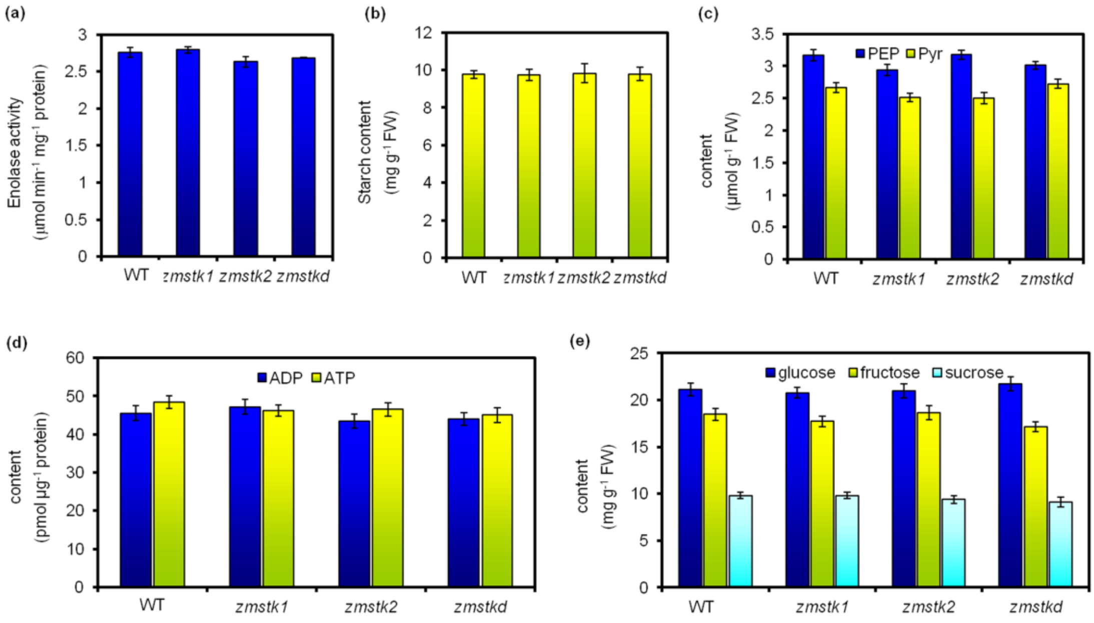

Figure S4 Activities of enolases and contents of metabolites in the immature ears. (a) Activities of enolases. (b) Content of starch. (c) Contents of PEP and pyruvate. (d) Contents of ADP and ATP. (e) Contents of glucose, sucrose, and fructose. Values are mean ± SE (n = 3).
