# Supplementary material for: ZmSTK1 and ZmSTK2, encoding receptor‐like cytoplasmic kinase, are involved in maize pollen development with additive effect
Source: Plant Biotechnol J. 2018 Feb 13;16(8):1402–14. doi: 10.1111/pbi.12880 (PMC6041449; doi:10.1111/pbi.12880)
Supplement: Supplementary file 2 — Table S1 The list of primers used in the study. [file PBI-16-1402-s001.pdf]

Table S1 The list of primers used in the present study

Gene	forward primer	reverse primer
**Ac insertion lines**		
AcP		CACACTGGCCAAAGGTTATCACA
ZmSTK1P	GCCGGCCAAAAATAGAGCTG(P1)	CCGTGTCGTGTCAAAGCATC(P2)
**Mu insertion lines**		
MuP		GCCTCYATTTCGTCGAATCC
ZmSTK2P	AAGCACAATGGGCAGGTACA(P3)	TGTGGTTGTCTTGAACCGCT(P4)
**cloning**		
Zm*STK1B*	ATGGGGCGGTCATACCTTGAA	AGCGTTCGACCTTCTCGGC
Zm*STK2B*	ATGGGCAGGTACAGCGACG	GTTGTAGTTGGACCTCCATGGA
**control genes**		
Actin	GTTGGGCGTCCTCGTCA	TGGGTCATCTTCTCCCTGTT
**qPCR**		
Zm*STK1C*	GCTTGACGGGCATGAAATGG	TGCTAGGCAGCTTATGCACC
Zm*STK2C*	AATCGCTAGCGTCAGGATCG	GCTTCTAATGCACGCAGAGC
**RNA Blot Analysis**		

Zm*STK1D*	AACAGGTACACGGCAATGGCAGAG	ACAGCAACATCTCACGGTCACACG
Zm*STK2D*	AGACGGGCATGCTGGGGGTGAAGT	ATGGCGGCGACGGGCTGGTGT
**Subcellular Localization**		
Zm*STK1E*	AAGCTTATGGGGCGGTCATACCTTGAA	GGATCCAGCGTTCGACCTTCTCGGC
Zm*STK2E*	AAGCTTATGGGCAGGTACAGCGACG	GGATCCGTTGTAGTTGGACCTCCATGGA
**Southern Blot Analysis**		
Zm*STK1D*	AACAGGTACACGGCAATGGCAGAG	ACAGCAACATCTCACGGTCACACG
Zm*STK2D*	AGACGGGCATGCTGGGGGTGAAGT	ATGGCGGCGACGGGCTGGTGT
